# Cochlea-sparing acoustic neuroma treatment with 4π radiation therapy

**DOI:** 10.1016/j.adro.2018.01.004

**Published:** 2018-02-05

**Authors:** Kaley Woods, Percy Lee, Tania Kaprealian, Isaac Yang, Ke Sheng

**Affiliations:** a Department of Radiation Oncology, University of California, Los Angeles, Los Angeles, California; b Department of Neurosurgery, University of California, Los Angeles, Los Angeles, California

## Abstract

**Purpose:**

This study investigates whether 4π noncoplanar radiation therapy can spare the cochleae and consequently potentially improve hearing preservation in patients with acoustic neuroma who are treated with radiation therapy.

**Methods and materials:**

Clinical radiation therapy plans for 30 patients with acoustic neuroma were included (14 stereotactic radiation surgery [SRS], 6 stereotactic radiation therapy [SRT], and 10 intensity modulated radiation therapy [IMRT]). The 4π plans were created for each patient with 20 optimal beams selected using a greedy column generation method and subsequently recalculated in Eclipse for comparison. Organ-at-risk (OAR) doses, homogeneity index, conformity, and tumor control probability (TCP) were compared. Normal tissue complication probability (NTCP) was calculated for sensorineural hearing loss (SNHL) at 3 and 5 years posttreatment. The dose for each plan was then escalated to achieve 99.5% TCP.

**Results:**

4π significantly reduced the mean dose to both cochleae by 2.0 Gy (32%) for SRS, 3.2 Gy (29%) for SRT, and 10.0 Gy (32%) for IMRT. The maximum dose to both cochleae was also reduced with 4π by 1.6 Gy (20%), 2.2 Gy (15%), and 7.1 Gy (18%) for SRS, SRT, and IMRT plans, respectively. The reductions in mean/maximum brainstem dose with 4π were also statistically significant. Mean doses to other OARs were reduced by 19% to 56% on average. 4π plans had a similar CN and TCP, with a significantly higher average homogeneity index (0.93 vs 0.92) and significantly lower average NTCP for SNHL at both 3 years (30.8% vs 40.8%) and 5 years (43.3% vs 61.7%). An average dose escalation of approximately 116% of the prescription dose achieved 99.5% TCP, which resulted in 32.6% and 43.4% NTCP for SNHL at 3 years and 46.4% and 64.7% at 5 years for 4π and clinical plans, respectively.

**Conclusions:**

Compared with clinical planning methods, optimized 4π radiation therapy enables statistically significant sparing of the cochleae in acoustic neuroma treatment as well as lowering of other OAR doses, potentially reducing the risk of hearing loss.

**Summary**

Radiation therapy of acoustic neuroma can cause hearing loss due to dose to the cochlear. 4π radiation therapy has been developed to maximally improve dose compactness and sparing of surrounding normal organs. In this study, 4π radiation therapy plans were created for 30 patients with acoustic neuroma and compared with conventional noncoplanar radiation therapy. 4π significantly reduced the mean and maximum dose to both cochleae, which can be correlated to reduced toxicity and potential dose escalation for patients with large tumors.

Alt-text: Unlabelled box

## Introduction

Acoustic neuroma, also known as vestibular schwannoma, is a benign brain tumor arising from the eighth cervical nerve. There are 2000 to 3000 new cases of benign acoustic neuroma diagnosed in the United States each year, approximately 25% of which are treated with radiation therapy.^1^ Due to its benign nature, the prognosis for patients with acoustic neuroma is typically very good, and with proper surveillance and treatment, no decrease in lifespan is expected. Therefore, the long-term posttreatment toxicity must be heavily weighted for these patients. Although the complication rates are much lower than with surgery,^2^, ^3^, ^4^ some patients experience radiation-induced side effects after treatment. Up to 40% of patients may experience middle ear side effects such as otitis media during treatment,^5^ which can cause tinnitus, dizziness, and pain. Almost half of patients may also experience some degree of sensorineural hearing loss (SNHL), which continues to worsen for years after treatment.^6^, ^7^, ^8^, ^9^, ^10^, ^11^, ^12^

There is evidence suggesting a correlation between the dose to the cochlea and the degree of hearing loss observed after radiation therapy for tumors in the head and neck region.^13^, ^14^, ^15^, ^16^, ^17^ For patients with acoustic neuroma who were treated with fractionated stereotactic radiation therapy, a study by Thomas et al showed that the minimum and maximum cochlear doses, as well as the percentage of the cochlea receiving 50%, 80%, and 90% of the prescription dose, were all strongly predictive of subsequent hearing deterioration.^18^ For stereotactic radiation surgery (SRS), significantly better hearing preservation was observed by Kano et al when the dose to the central cochlea was kept below 4.2 Gy.^19^ Timmer et al also demonstrated a correlation between the maximum cochlear dose and the extent of hearing loss in patients with acoustic neuroma who were treated with Gamma Knife radiation surgery.^20^

In addition to hearing loss, many patients with acoustic neuroma experience cranial neuropathy after radiation therapy. In a study of 149 cases of acoustic neuroma radiosurgery, Foote et al found that the maximum dose to the brainstem was the most significant predictor of the incidence of facial, trigeminal, or any other type of neuropathy after treatment.^21^ Therefore, the sparing of dose to the brainstem must also be a high priority for these patients.

However, adequate radiation doses must still be delivered to achieve long-term tumor control. A large Gamma Knife patient cohort established that a median single-fractional dose of 13 Gy to the tumor margin (50% isodose) is necessary for local control.^8^ This dose prescription typically results in maximum doses of 26 Gy for Gamma Knife plans, which may not always be safely deliverable,^22^ particularly for larger tumors. Although 12 to 13 Gy is also the standard prescription dose for linac-based single-fraction acoustic neuroma treatment, these plans follow different prescription conventions (typically 100% of the prescription dose to 95%-100% of the target volume) and result in more homogeneous dose distributions with lower maximum doses.

Therefore, to reduce the risk of hearing loss and other normal tissue complications after treatment while also delivering enough dose for maximal tumor control, highly conformal dose distributions are needed that can better spare the surrounding normal tissue. The dosimetry of Gamma Knife has been compared with conformal and dynamic arcs using linacs for acoustic neuroma treatment.^23^ Although the Gamma Knife dose was slightly more conformal (by 2%), the maximal dose was also much higher. This may help local control but can increase the risk of hearing loss and other neurologic side effects because the intracanalicular component of the cochlear nerve, the cochlear ramus of the internal auditory artery, and the facial nerve all traverse the target volume.^24^

Another advantage of linac-based treatment is that the treatment can be fractionated for larger tumors. A clinically relevant question is whether recent advances in treatment planning techniques can be used to further improve linac plans. 4π radiotherapy, with optimized noncoplanar beam orientations, has been shown to significantly reduce normal tissue doses in the liver, prostate, brain, lung, and head and neck.^25^, ^26^, ^27^, ^28^ The aim of this study is to determine whether 4π can also produce superior dosimetry for acoustic neuroma treatment, potentially providing better sparing of the cochleae and reducing the risk of radiation-induced complications such as hearing loss.

## Methods and materials

### Clinical plans

Thirty patients who were previously treated with radiation therapy for benign acoustic neuroma were included in this study, and their computed tomography images, plan, dose, and contours were obtained (Table 1). Fourteen of these patients were treated with single-fraction SRS and prescription doses of 12 to 13 Gy. Six patients received stereotactic radiation therapy (SRT) with 5 fractions of 5 Gy each. Ten patients received intensity modulated radiation therapy (IMRT) with 28 to 30 fractions of 1.8 Gy each. Static beams IMRT (7-11 beams) were used for 13 patients, dynamic conformal arcs (4-5 partial noncoplanar arcs) were used for 11 patients, and volumetric-modulated arcs (2 full coplanar arcs or 2-4 noncoplanar partial arcs) were used for 6 patients.

**Table 1:** Patient data

	Prescription dose (Gy)	Fractions	Plan type	PTV volume (cm^3^)		Prescription dose (Gy)	Fractions	Plan type	PTV volume (cm^3^)
**1**	12	1	DCAT	0.5	**16**	25	5	Static IMRT	1.07
**2**	12	1	Static IMRT	5.48	**17**	25	5	Static IMRT	2.45
**3**	12	1	Static IMRT	2.7	**18**	25	5	VMAT	8.12
**4**	12	1	DCAT	1.66	**19**	25	5	Static IMRT	0.24
**5**	12	1	DCAT	3.33	**20**	25	5	Static IMRT	0.17
**6**	12	1	DCAT	2.75	**21**	50.4	28	VMAT	6.3
**7**	12	1	Static IMRT	0.74	**22**	50.4	28	VMAT	35.81
**8**	12	1	DCAT	2.79	**23**	50.4	28	Static IMRT	17.29
**9**	12	1	Static IMRT	2.54	**24**	50.4	28	DCAT	0.35
**10**	12	1	DCAT	2.24	**25**	50.4	28	DCAT	2.42
**11**	12	1	Static IMRT	3.1	**26**	50.4	28	DCAT	0.92
**12**	12	1	Static IMRT	5.23	**27**	50.4	28	VMAT	10.87
**13**	12	1	DCAT	2.65	**28**	50.4	28	Static IMRT	2.78
**14**	13	1	DCAT	1.31	**29**	50.4	28	VMAT	13.58
**15**	25	5	Static IMRT	2.54	**30**	54	30	VMAT	23.22

DCAT, dynamic conformal arc therapy; IMRT, intensity modulated radiation therapy; PTV, planning target volume; VMAT, volumetric-modulated arc therapy.

**Figure 1:**
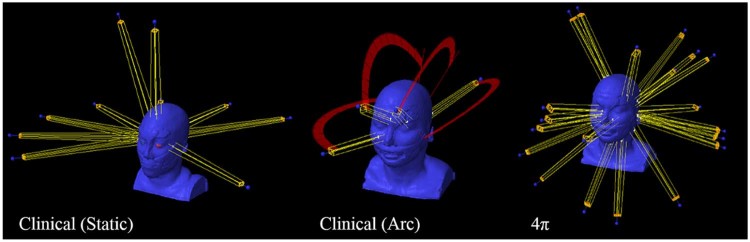
Examples of typical beam orientations for the clinical plans with 7 to 11 static beams (left), clinical plans with 2 to 5 arcs (middle), and 4π plans with 20 static beams (right). From Table 1, these are patients #16, #4, and #1, respectively.

The plans were created using the machine parameter file for a Novalis Tx machine equipped with a 0.25 cm high-definition, multileaf collimator. The dose calculation resolution was 2 mm using the Analytical Anisotropic Algorithm, Version 10.0.28. The treatment regimen was determined on the basis of tumor size and achievable organ-at-risk (OAR) sparing. Examples of the beam orientations for these plans are shown in Figure 1. The brainstem, chiasm, cochlea (one or both, depending on the tumor location), eyes, lenses, and optical nerves were included as critical organs for all plans.

### 4π plans

4π plans were made for each patient using the optimization process previously published.^25^, ^29^ The optimization process started with a pool of 1162 candidate beams making up the 4π solid angle space, each with a separation of 6°. A computer-assisted design model of the Varian TrueBeam system, along with a 3-dimensional patient model, was used to detect any potential collisions between the gantry and the couch or patient.

After these beams were eliminated, the dose was calculated for 5 × 5 mm^2^ beamlets using convolution/superposition with a 6 MV polyenergetic kernel. Subsequently, a greedy column generation method was used to perform an integrated beam orientation and fluence map optimization.^29^ The objective function includes manually tuned parameters for the priority and weighting of each OAR, which were set to penalize dose to the cochlea and other nearby critical organs while maintaining planning target volume (PTV) coverage. A final beam count of 20 was chosen to fully exploit the noncoplanar space while maintaining reasonable deliverability, as shown by a prospective patient study.^30^

Next, using the optimized beams, fluence map optimization and dose calculation were performed again in Eclipse with parameters that were identical to those of the clinical plans, for an unbiased comparison. The planning goal was to match the PTV coverage of the clinical plans while reducing the dose to the cochleae and reducing or maintaining the doses to all other OARs.

### Plan comparison

The 4π and clinical plans were evaluated on the basis of their PTV coverage and normal tissue sparing, specifically of the cochleae. The mean and maximum doses for the cochleae and all other OARs were compared for all plans. The volume receiving 50% of the prescription dose (V50%) was calculated to evaluate the dose spillage for each plan. To evaluate dose conformity, R_100_ (the ratio of the 100% isodose volume to the PTV) was compared, along with the van't Riet conformation number, defined as:

(1) $$CN=\frac{V_{T,Rx}}{V_{T}}\times \frac{V_{T,Rx}}{V_{Rx}}$$

where *V_T_* is the target volume, *V_T_*_,_*_Rx_* is the volume of the target receiving a dose equal to or greater than the prescription dose, and *V_Rx_* is the total volume receiving the prescription dose.^31^ The homogeneity index was also calculated as:

(2) $$HI=1+\frac{\left(D2\%-D98\%\right)}{D_{Rx}}$$

where *D_Rx_* is the prescription dose and *D*2% and *D*98% are the minimum doses to 2% and 98% of the PTV volume.^32^

Radiobiologic modeling was also used to predict the tumor control probability (TCP) and normal tissue complication probability (NTCP) for SNHL. The definition of SNHL differs between studies but is typically considered a loss of at least 10 to 20 dB in one or more frequencies. Because hearing function has been shown to continue deteriorating over long follow-up times,^9^ the SNHL NTCP at both 3 and 5 years posttreatment was calculated for each plan.

The TCP was calculated using the Poisson-based model with the parameters shown in Table 2. The cochlea effective volume (V_eff_) was calculated with the Kutcher-Burman dose volume histogram reduction scheme, which estimated the volume of the cochlea and, if homogenously irradiated to the prescription dose, would result in the same NTCP as the actual inhomogeneous dose distribution.^33^ This effective volume was then used to predict the NTCP values for the cochlea with the Lyman model,^34^ using the parameters in Table 2.

**Table 2:** TCP and NTCP model parameters

Model parameter^a^	TCP	SNHL NTCP (3 years)	SNHL NTCP (5 years)
**α/β**	2.4 Gy	2 Gy	2 Gy
**TCD_50_**	27 Gy	–	–
**TD_50_**	–	31.5 Gy	19.25 Gy
**γ_50_**	1.5	0.71	0.46
**n**	–	0.83	0.83

γ_50_, slope of sigmoidal dose response curve at 50% tumor control probability/normal tissue complication probability; n, volume-effect parameter; TCD_50_, tumor dose to achieve 50% tumor control probability; TD_50_, whole organ dose resulting in 50% normal tissue complication probability; TCP, tumor control probability; NTCP, normal tissue complication probability; SNHL, sensorineural hearing loss.

a α/β: Ratio of the linear and quadratic terms of the organ-specific dose response curve.

All model parameters were selected on the basis of published clinical data on the relationship between treatment outcomes (tumor control and complication rates) and dose delivered to the tumor and cochleae.^9^, ^10^, ^11^, ^12^ Because the fractionation schemes varied widely among patients in this study, all plan doses were normalized to a reference dose of 2 Gy per fraction for radiobiologic modeling.

### Dose escalation

Although excellent local control rates have been reported for acoustic neuroma treated with SRS and SRT, the control rates decrease in long-term follow up, particularly for larger tumors.^12^, ^35^ Therefore, dose escalation may be beneficial if normal tissue complication rates remain low. A dose escalation study was performed to evaluate whether 4π radiation therapy could achieve higher tumor control rates without increasing the risk of hearing loss for patients with acoustic neuroma. The prescription doses for each plan were escalated until the plans achieved TCP values of 99.5%. The NTCP values for SNHL at 3 and 5 years posttreatment were calculated for the escalated dose distributions and compared for the clinical and 4π plans.

## Results

### Plan comparison

**Figure 2:**
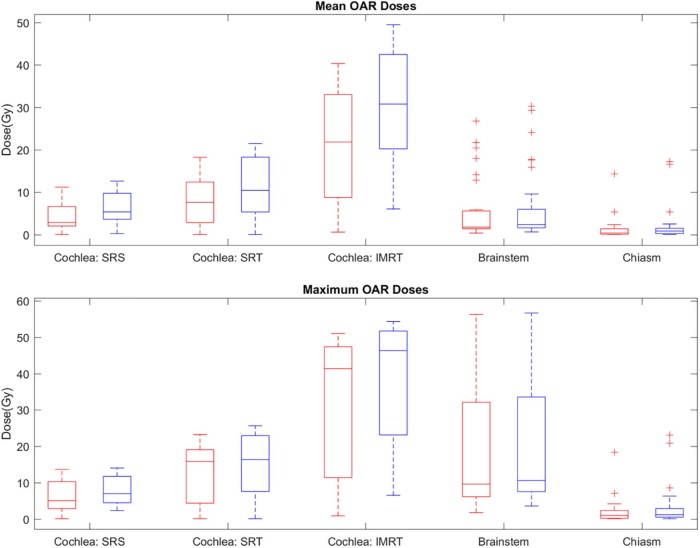
Mean (top) and maximum (bottom) doses to the cochlea (stereotactic radiation surgery, stereotactic radiation therapy, and intensity modulated radiation therapy groups), brainstem, and chiasm with the 4π plans (red) and clinical plans (blue). All differences between the 4π and clinical plans were statistically significant (2-tailed *t* test, 5% significance level), except for the maximum dose to the chiasm.

The OAR doses and conformity measures for both plan types are given in Table 3 and Figure 2. The mean cochlear dose was significantly reduced with 4π from 6.29 to 4.25 Gy for SRS plans, from 11.20 to 8.00 Gy for SRT plans, and from 30.88 to 20.93 Gy for IMRT plans. The maximum cochlear dose was also significantly reduced by 1.58 (20%), 2.2 (15%), and 7.1 Gy (18%) for SRS, SRT, and IMRT, respectively.

**Table 3:** OAR doses and conformity measures for both plan types

Plan type		Average OAR doses (Gy)					V50% (cm^3^)	PTV HI	van't RietCN
		Brainstem	Chiasm	Cochleae					
				SRS	SRT	IMRT			
**Clinical**	Mean	6.61	2.12	6.29	11.20	30.88	25.23	0.92	0.69
	Max	20.03	3.27	8.05	14.92	38.79			
**4π**	Mean	5.41^a^	1.30^a^	4.25^a^	8.00^a^	20.93^a^	24.85	0.93^a^	0.73
	Max	18.59^a^	2.04	6.47^a^	12.72^a^	31.74^a^			

CN, conformation number; HI, homogeneity index; IMRT, intensity modulated radiation therapy; max, maximum; OAR, organ at risk; SRS, stereotactic radiation surgery; SRT, stereotactic radiation therapy; PTV, planning target volume; V50%, volume that received 50% of the prescription dose.

a Statistically significant difference from the clinical plans (2-tailed *t* test; *p* < .05).

**Figure 3:**
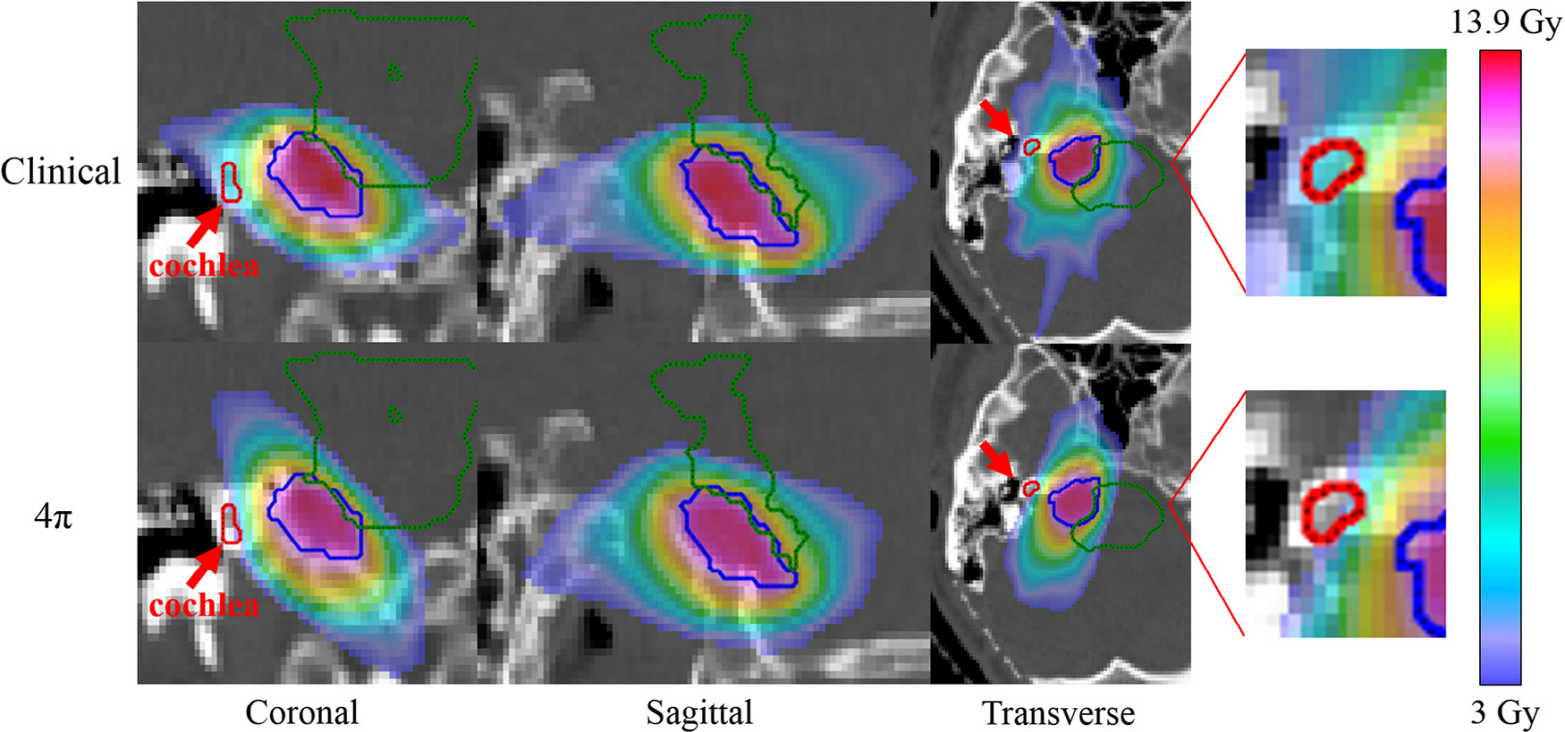
Dose color wash for a patient (patient #3 in Table 1) who was treated to a prescription dose of 12 Gy in a single fraction. Structures: Planning target volume (blue), brainstem (green), and cochlea (red).

In addition, there was significant sparing of the brainstem with 4π, which reduced the mean and maximum doses by 18% and 7%, respectively. These reductions were achieved with a steeper dose gradient around the target, as illustrated by the isodose colorwash in Figure 3. The mean and maximum doses to the chiasm were also 39% and 38% lower, respectively, for the 4π plans than for the clinical plans. The mean doses to the eyes, lenses, and optical nerves were reduced by 19% to 56% on average with 4π.

**Figure 4:**
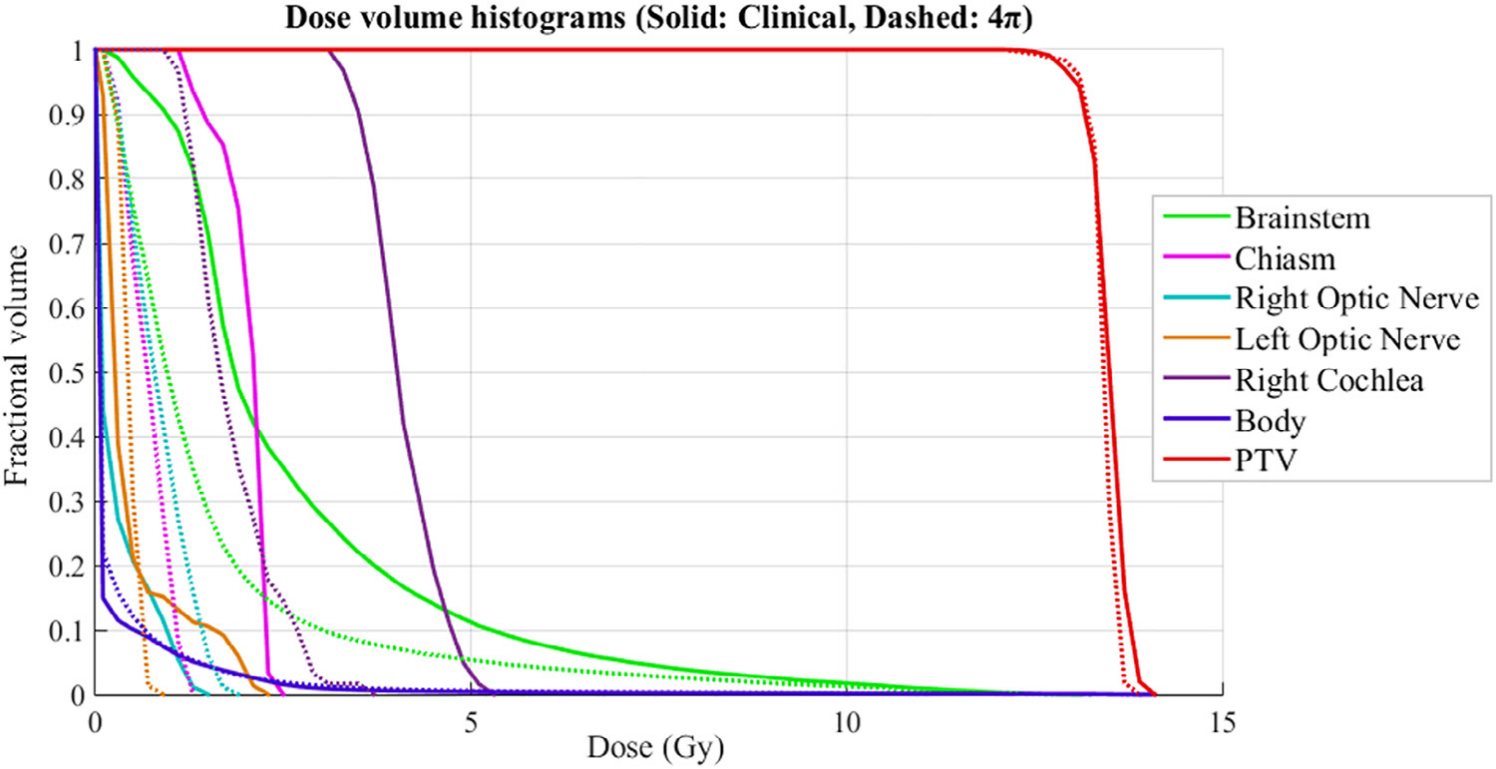
Dose volume histograms (solid line: clinical plan; dashed line: 4π plan) for one of the patients who underwent stereotactic radiation surgery in this study (patient #2 in Table 1), with a prescription dose of 12 Gy.

The conformity measures were also slightly better for the 4π plan. The average V50% was 0.38 cm^3^ lower for the 4π plans than for the clinical plans. The 4π plans were also able to maintain similar PTV coverage despite the major reductions in OAR doses, as illustrated in the dose volume histogram in Figure 4. The average R_100_ ratio was better for the 4π plans (1.32 vs 1.41) as well as the van't Riet conformation number (0.73 vs 0.69). There was also a statistically significant increase in the PTV homogeneity index with 4π (0.93 vs 0.92). The total monitor units for each plan were 2248 for the 4π plans, on average, and 1561 for the clinical plans.

4π consistently improved the cochlea sparing compared with IMRT, dynamic conformal arc, and volumetric-modulated arc therapy (VMAT) patient subcohorts. The mean cochlea dose was reduced by 32.2%, 26.8%, and 35.1%, respectively. The maximum cochlea dose was reduced by 19.4%, 11.3%, and 21.3%, respectively.

The results of the radiobiologic modeling study are given in Table 4. Although the difference was statistically significant, the TCP for the clinical plans was only 0.3% higher for the clinical plans than for the 4π plans. This difference can be explained by the significantly greater hotspots within the PTV for the clinical plans because all plans were normalized for the same target coverage. The estimated TCP is consistent with clinical reports, in which prescription doses of at least 12 Gy for SRS and 50 Gy for IMRT yielded TCP values of >90%.^9^, ^10^, ^11^, ^12^ All NTCP predictions for SNHL were significantly higher for the clinical plans than for the 4π plans. The average NTCP was 10.0% higher for the clinical plans at 3 years and 18.4% higher at 5 years posttreatment.

**Table 4:** Results of the radiobiological modeling and dose escalation study

Plan type	Average TCP	Average SNHL NTCP		Average escalation factor	Escalated SNHL NTCP	
		3 years	5 years		3 years	5 years
**Clinical**	95.7 ± 0.9%	40.8 ± 5.9%	61.7 ± 10.8%	1.162 ± 0.02	43.4 ± 6.3%	64.7 ± 11.1%
**4π**	95.4 ± 0.9%^a^	30.8 ± 5.3%^a^	43.3 ± 11.2%^a^	1.166 ± 0.02^a^	32.6 ± 5.5%^a^	46.4% ± 11.3^a^

SNHL, sensorineural hearing loss; NTCP, normal tissue complication probability; TCP, tumor control probability.

a Statistically significant difference from the clinical plans (2-tailed *t* test; *p* < .05; 95% confidence intervals).

### Dose escalation

To achieve a TCP of 99.5%, the prescription doses had to be escalated by a factor of approximately 1.16 on average for both plan types. After dose escalation, the probabilities of posttreatment SNHL at 3 and 5 years were once again significantly lower for the 4π plans, by 10.8% and 18.3%, respectively. The average NTCP for the escalated 4π plans was 8.2% lower than the normal (nonescalated) clinical plans at 3 years and 15.4% lower at 5 years posttreatment.

### Discussion

Although normal tissue complications are a concern for every patient receiving radiation therapy, these risks must be weighted even more heavily for the treatment of benign tumors such as acoustic neuroma. The majority of these patients will have an unaffected life expectancy, and any radiation-induced side effects could have a lasting impact on quality of life that competes with the benefits of treatment. In addition, acoustic neuroma is a highly treatable disease. Therefore, despite high treatment success rates, technologic improvements should not stop until 100% local control is achieved because uncontrolled tumor growth may ultimately compromise patient hearing and other neurologic functions. Although excellent local control (>98%) can be achieved with surgical resection, this treatment option carries significant risks in addition to hearing loss. More than 20% of surgical patients experience complications such as facial paralysis, neurologic damage, cerebrospinal fluid fistula, hematoma, hydrocephalus, and even death.^2^, ^3^, ^4^

Radiation surgery is increasingly chosen over surgery as a noninvasive treatment option but there are still a significant number of cases in which the treatment either fails to control the tumor growth or the tumor eventually recurs. Surgical resection is typically the next course of action in these cases because further radiation therapy would exceed normal tissue dose tolerances. In a study by Yomo et al of repeat radiation surgery for patients with acoustic neuroma, 2 patients required as many as 3 Gamma Knife treatments (with prescription doses of 12, 12, and 14 Gy) before achieving tumor control.^36^ None of the patients in this study maintained useful hearing after receiving repeat radiation surgery. Although an initial target dose of 12 Gy was clearly insufficient for achieving or maintaining tumor regression in these patients, the delivery of larger single-fraction doses is typically limited by normal tissue tolerances.

When using noncoplanar conformal and dynamic arcs on a modern linac, dose conformity that is nearly as good as that with Gamma Knife can be achieved, without the toxic high maximal doses.^23^, ^24^ As demonstrated in this study, 4π radiation therapy can enable statistically significant reductions in both mean and maximum normal tissue doses, particularly to the cochlea and brainstem. As previously mentioned, clinical studies have found that patients who receive <4.2 Gy single fractional doses to the cochlea experience significantly better hearing preservation than patients who receive a > 4.2 Gy dose.

Our study shows that for patients treated with a single fractional SRS dose, the mean cochlear dose can be reduced from 6.29 Gy to 4.25 Gy, allowing potentially significant reductions in hearing loss. On the other hand, we showed that prescription doses for the 4π plans can be escalated to achieve 99.5% tumor control while maintaining hearing loss probabilities below the nonescalated clinical plans. The ability to safely escalate prescription doses would likely reduce the incidence of tumor recurrence and the need for subsequent tumor resection or secondary radiation, both of which carry major risks of hearing loss or other complications.

In this study, heterogeneous planning techniques including conformal arc, IMRT, and VMAT were used clinically, mainly depending on the size of the tumor. Conformal arcs were mainly used on smaller tumors, and larger tumors benefit from the better dose homogeneity and conformity that is achieved with intensity modulation in IMRT and VMAT. Nevertheless, 4π radiation therapy resulted in consistently improved cochlea sparing in individual planning technique comparisons.

In this study, the noncoplanar beams were not actually delivered. In a separate prospective clinical study,^30^ a similar number of beams was delivered to patients with brain tumors. In this study, the beams were ordered on the basis of their couch rotation angles. That way, the treatment could be delivered in a single couch sweep while the gantry rotated back and forth under the guidance of pretreatment modeling.^37^ With remote manual machine operation, the treatment delivery time was less than 35 minutes. In the phantom test using fully automated machine control that has not been approved for patients, the same treatment could be delivered in less than 15 minutes. We expect similar treatment time for patients with acoustic neuroma.

## Conclusions

4π radiotherapy achieves significantly greater normal tissue sparing compared with radiation therapy techniques that are typically used in acoustic neuroma treatment. These major reductions in cochlear dose may reduce the risk of normal tissue complications such as hearing loss and enable the safe escalation of prescription doses to potentially improve tumor control rates.

## References

[1] Bisi M.A., Selaimen C.M., Chaves K.D., Bisi M.C., Grossi M.L. (2006). Vestibular schwannoma (acoustic neuroma) mimicking temporomandibular disorders: A case report. J Appl Oral Sci.

[2] Kaylie D.M., Gilbert E., Horgan M.A., Delashaw J.B., McMenomey S.O. (2001). Acoustic neuroma surgery outcomes. Otol Neurotol.

[3] Patel S., Nuno M., Mukherjee D. (2012). Trends in surgical use and associated patient outcomes in the treatment of acoustic neuroma. World Neurosurg.

[4] Darrouzet V., Martel J., Enee V., Bebear J.P., Guerin J. (2004). Vestibular schwannoma surgery outcomes: Our multidisciplinary experience in 400 cases over 17 years. Laryngoscope.

[5] Jereczek-Fossa B.A., Zarowski A., Milani F., Orecchia R. (2003). Radiotherapy-induced ear toxicity. Cancer Treat Rev.

[6] Combs S.E., Engelhard C., Kopp C. (2015). Long-term outcome after highly advanced single-dose or fractionated radiotherapy in patients with vestibular schwannomas—pooled results from 3 large German centers. Radiother Oncol.

[7] De Marzi L., Feuvret L., Boulé T. (2015). Use of gEUD for predicting ear and pituitary gland damage following proton and photon radiation therapy. Br J Radiol.

[8] Lunsford L.D., Niranjan A., Flickinger J.C., Maitz A., Kondziolka D. (2005). Radiosurgery of vestibular schwannomas: Summary of experience in 829 cases. J Neurosurg.

[9] Hasegawa T., Kida Y., Kato T., Iizuka H., Yamamoto T. (2011). Factors associated with hearing preservation after Gamma Knife surgery for vestibular schwannomas in patients who retain serviceable hearing. J Neurosurg.

[10] Ikonomidis C., Pica A., Bloch J., Maire R. (2015). Vestibular schwannoma: The evolution of hearing and tumor size in natural course and after treatment by LINAC stereotactic radiosurgery. Audiol Neurootol.

[11] Roos D.E., Potter A.E., Zacest A.C. (2011). Hearing preservation after low dose linac radiosurgery for acoustic neuroma depends on initial hearing and time. Radiother Oncol.

[12] Milligan B.D., Pollock B.E., Foote R.L., Link M.J. (2012). Long-term tumor control and cranial nerve outcomes following gamma knife surgery for larger-volume vestibular schwannomas. J Neurosurg.

[13] van der Putten L., de Bree R., Plukker J.T. (2006). Permanent unilateral hearing loss after radiotherapy for parotid gland tumors. Head Neck.

[14] Chen W.C., Jackson A., Budnick A.S. (2017). Sensorineural hearing loss in combined modality treatment of nasopharyngeal carcinoma. Cancer.

[15] Bhandare N., Antonelli P.J., Morris C.G., Malayapa R.S., Mendenhall W.M. (2007). Ototoxicity after radiotherapy for head and neck tumors. Int J Radiat Oncol Biol Phys.

[16] Hua C., Bass J.K., Khan R., Kun L.E., Merchant T.E. (2008). Hearing loss after radiotherapy for pediatric brain tumors: Effect of cochlear dose. Int J Radiat Oncol Biol Phys.

[17] Pan C.C., Eisbruch A., Lee J.S., Snorrason R.M., Ten Haken R.K., Kileny P.R. (2005). Prospective study of inner ear radiation dose and hearing loss in head-and-neck cancer patients. Int J Radiat Oncol Biol Phys.

[18] Thomas C., Di Maio S., Ma R. (2007). Hearing preservation following fractionated stereotactic radiotherapy for vestibular schwannomas: Prognostic implications of cochlear dose. J Neurosurg.

[19] Kano H., Kondziolka D., Khan A., Flickinger J.C., Lunsford L.D. (2009). Predictors of hearing preservation after stereotactic radiosurgery for acoustic neuroma. J Neurosurg.

[20] Timmer F.C., Hanssens P.E., van Haren A.E. (2016). Gamma knife radiosurgery for vestibular schwannomas: Results of hearing preservation in relation to the cochlear radiation dose. Laryngoscope.

[21] Foote K.D., Friedman W.A., Buatti J.M., Meeks S.L., Bova F.J., Kubilis P.S. (2001). Analysis of risk factors associated with radiosurgery for vestibular schwannoma. J Neurosurg.

[22] Arthurs B.J., Lamoreaux W.T., Giddings N.A. (2009). Gamma Knife radiosurgery for vestibular schwannoma: Case report and review of the literature. World J Surg Oncol.

[23] Perks J.R., St George E.J., El Hamri K., Blackburn P., Plowman P.N. (2003). Stereotactic radiosurgery XVI: Isodosimetric comparison of photon stereotactic radiosurgery techniques (Gamma Knife vs. micromultileaf collimator linear accelerator) for acoustic neuroma–and potential clinical importance. Int J Radiat Oncol Biol Phys.

[24] Plowman P.N., Doughty D. (1999). Stereotactic radiosurgery, X: Clinical isodosimetry of Gamma Knife versus linear accelerator X-knife for pituitary and acoustic tumours. Clin Oncol (R Coll Radiol).

[25] Dong P., Lee P., Ruan D. (2012). 4pi non-coplanar liver SBRT: A novel delivery technique. Int J Radiat Oncol Biol Phys.

[26] Dong P., Nguyen D., Ruan D. (2014). Feasibility of prostate robotic radiation therapy on conventional C-arm linacs. Pract Radiat Oncol.

[27] Dong P., Lee P., Ruan D. (2013). 4pi noncoplanar stereotactic body radiation therapy for centrally located or larger lung tumors. Int J Radiat Oncol Biol Phys.

[28] Rwigema J.C., Nguyen D., Heron D.E. (2014). 4pi noncoplanar stereotactic body radiation therapy for head-and-neck cancer: Potential to improve tumor control and late toxicity. Int J Radiat Oncol Biol Phys.

[29] Romeijn H.E., Ahuja R.K., Dempsey J.F., Kumar A. (2005). A column generation approach to radiation therapy treatment planning using aperture modulation. SIAM J Optim.

[30] Yu V.Y., Landers A., Woods K. (2018). A prospective 4pi radiotherapy clinical study in recurrent high grade glioma patients. Int J Radiat Oncol Biol Phys.

[31] van't Riet A., Mak A.C., Moerland M.A., Elders L.H., van der Zee W. (1997). A conformation number to quantify the degree of conformality in brachytherapy and external beam irradiation: Application to the prostate. Int J Radiat Oncol Biol Phys.

[32] Sheng K., Molloy J.A., Larner J.M., Read P.W. (2007). A dosimetric comparison of non-coplanar IMRT versus helical tomotherapy for nasal cavity and paranasal sinus cancer. Radiother Oncol.

[33] Kutcher G.J., Burman C. (1989). Calculation of complication probability factors for non-uniform normal tissue irradiation: The effective volume method. Int J Radiat Oncol Biol Phys.

[34] Lyman J.T. (1985). Complication probability as assessed from dose-volume histograms. Radiat Res.

[35] Watanabe S., Yamamoto M., Kawabe T. (2016). Stereotactic radiosurgery for vestibular schwannomas: Average 10-year follow-up results focusing on long-term hearing preservation. J Neurosurg.

[36] Yomo S., Arkha Y., Delsanti C., Roche P.H., Thomassin J.M., Regis J. (2008). Repeat Gamma Knife surgery for regrowth of vestibular schwannomas. Neurosurgery.

[37] Yu V.Y., Tran A., Nguyen D. (2015). The development and verification of a highly accurate collision prediction model for automated noncoplanar plan delivery. Med Phys.

